# Kissing balloon technique as rescue strategy to treat left ventricular outflow tract obstruction after transcatheter mitral valve replacement: a case report

**DOI:** 10.1093/ehjcr/ytaf021

**Published:** 2025-01-22

**Authors:** Sibel Çatalkaya, Hakan Erkan

**Affiliations:** Department of Cardiology, Bursa City Hospital, Doğanköy Mahallesi Şifa Caddesi No:1 16250 Nilüfer/Bursa, Turkey; Department of Cardiology, Bursa City Hospital, Doğanköy Mahallesi Şifa Caddesi No:1 16250 Nilüfer/Bursa, Turkey

**Keywords:** Case report, Transcatheter mitral valve replacement, Mitral stenosis, Left ventricular outflow tract obstruction, Kissing balloon technique

## Abstract

**Background:**

Transcatheter mitral valve replacement (TMVR) has become a viable, minimally invasive treatment for inoperable patients with severe mitral valve disease, particularly among elderly individuals with significant comorbidities. A key complication of TMVR is left ventricular outflow tract (LVOT) obstruction, necessitating various preventive and therapeutic strategies. This report presents a case of severe LVOT obstruction following TMVR and highlights the effective application of the kissing balloon technique as a therapeutic intervention.

**Case summary:**

A 79-year-old female with New York Heart Association Class IV dyspnoea due to severe mitral stenosis and a high operative risk, with a Society of Thoracic Surgeons Risk of Mortality (STS-PROM) score of 6.2%, underwent TMVR. Pre-procedural evaluations indicated significant mitral valve calcification and a mitral valve area of 0.9 cm². After successful TMVR deployment, post-implantation echocardiography revealed an LVOT pressure gradient of 53/85 mmHg, prompting the use of the kissing balloon technique, which reduced the gradient to 28 mmHg. Follow-up assessments showed normal mitral valve function and stable LVOT gradients during short-term follow-up throughout the patient’s hospital stay.

**Discussion:**

Left ventricular outflow tract obstruction is a potentially life-threatening complication of TMVR, often associated with high mortality rates due to haemodynamic impairment. This complication can arise from various anatomical factors and valve positioning issues. Several strategies have been developed to address LVOT obstruction, including the laceration of the anterior mitral leaflet and alcohol septal ablation. The successful implementation of the kissing balloon technique in this case underscores its potential to improve outcomes in LVOT obstruction.

**Learning points**

- To understand the causes of left ventricular outflow tract obstruction after transcatheter mitral valve replacement.
- To demonstrate that the kissing balloon technique can be successfully applied as a rescue strategy in patients with left ventricular outflow tract obstruction following transcatheter mitral valve replacement.

## Introduction

Transcatheter mitral valve replacement (TMVR) has emerged as a minimally invasive therapeutic option for inoperable patients with severe mitral valve disease. While this approach offers considerable advantages, it also presents a range of technical and clinical challenges, particularly in elderly patients with significant comorbidities. A critical concern associated with TMVR is left ventricular outflow tract (LVOT) obstruction, for which various preventive and therapeutic strategies have been developed. In this report, we present a case of severe LVOT obstruction following TMVR and illustrate the successful application of the kissing balloon technique as a valuable therapeutic option.

## Summary figure

**Figure:**
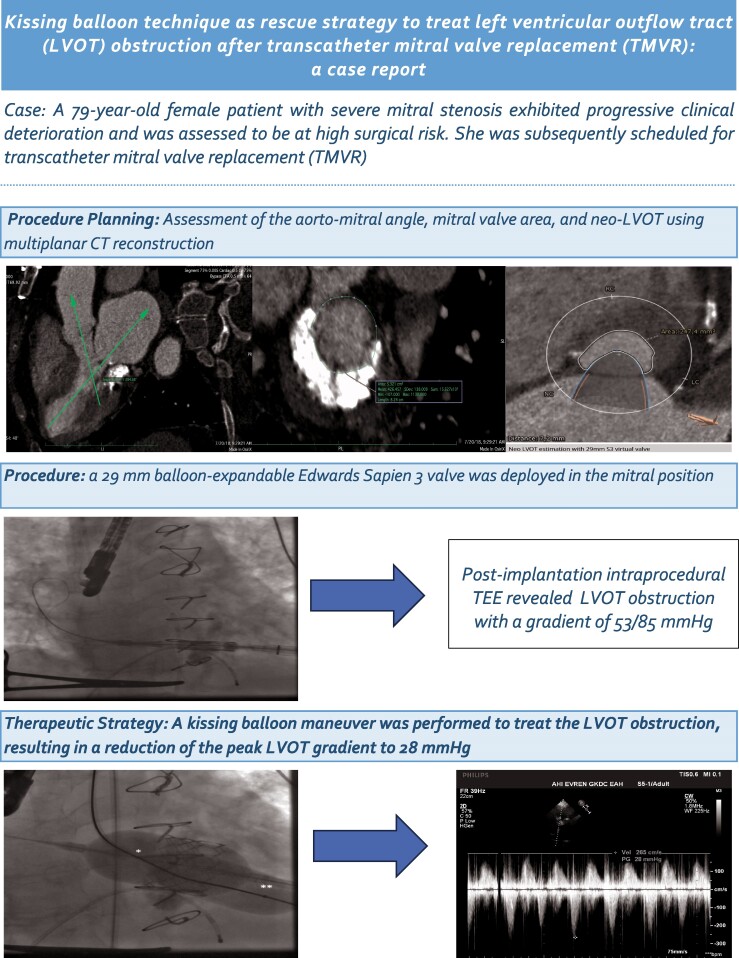


## Case presentation

A 79-year-old female patient with progressive dyspnoea (New York Heart Association functional Class IV) was referred to our centre to assess her eligibility for mitral valve surgery due to severe mitral stenosis. Her medical history included coronary artery disease with coronary bypass grafting, heart failure with reduced systolic left ventricular function, atrial fibrillation, arterial hypertension, and lymphoma in remission after radiotherapy. Her pharmacological therapy included a beta-blocker (metoprolol), angiotensin-converting enzyme inhibitor (ramipril), aldosterone antagonist (spironolactone), diuretic therapy (furosemide), and vitamin K antagonist (warfarin).

**Figure 1:**
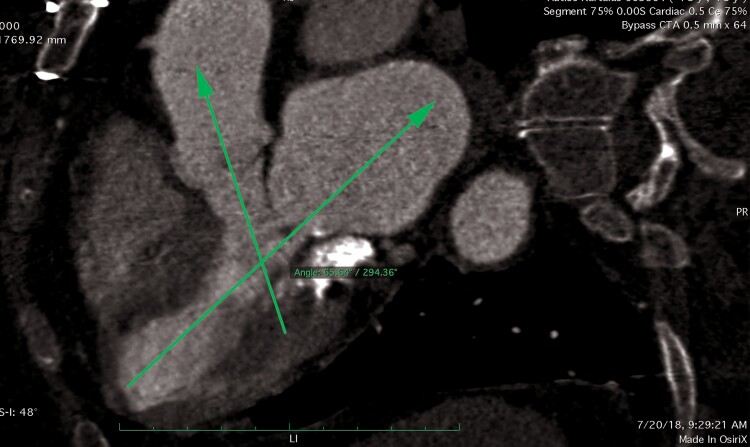
Aorto-mitral angle was measured 65/294° on multiplanar cardiac-gated computed tomography.

**Figure 2:**
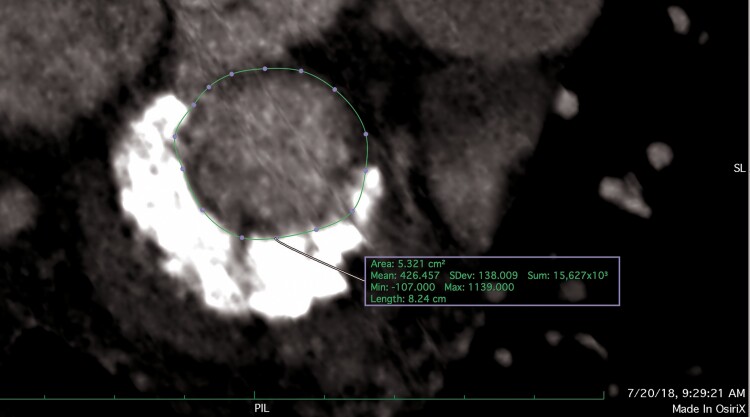
Assessment of mitral valve area for transcatheter mitral valve replacement on multiplanar cardiac-gated computed tomography.

**Figure 3:**
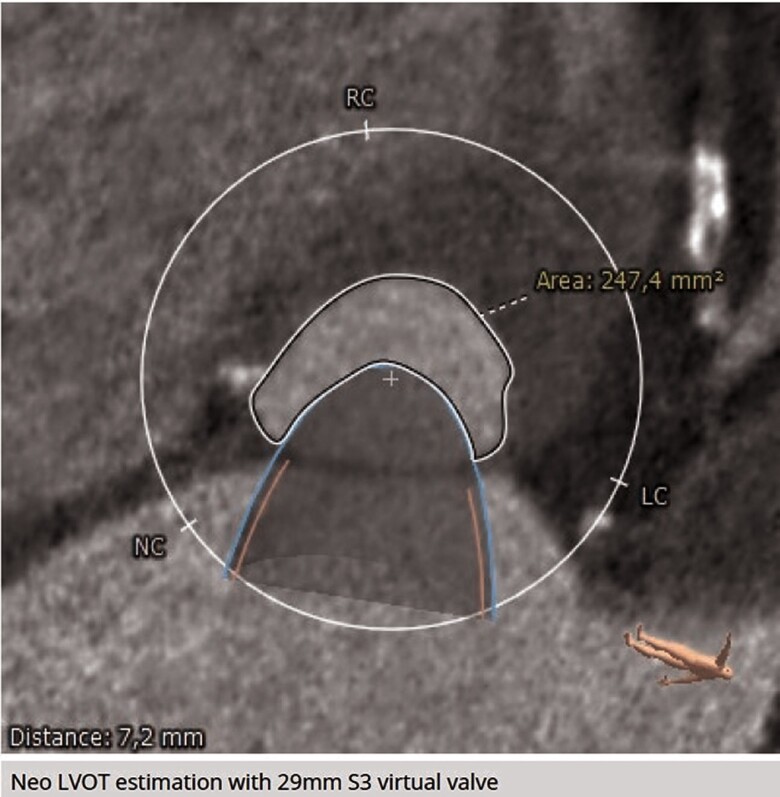
Virtual estimation of neo-left ventricular outflow tract area for the Edwards SAPIEN 3 valve on multiplanar cardiac-gated computed tomography.

Upon admission, the patient presented in a frail general condition with stable vital signs, including a blood pressure of 115/65 mmHg, a heart rate of 89 b.p.m., and an oxygen saturation of 90% on room air. Physical examination revealed a rumbling diastolic murmur at the apex, crackles over the basal lung fields, and peripheral oedema. Echocardiographic assessment showed reduced systolic left ventricular function with an ejection fraction of 35%, severe mitral stenosis, and moderate tricuspid regurgitation, with a peak tricuspid regurgitation velocity of 3.8 m/s, corresponding to a peak tricuspid regurgitation gradient of 58 mmHg. There was severe calcification of the mitral valve apparatus with significant mitral annular calcification, particularly affecting the posterior annulus fibrosus. The posterior mitral leaflet was immobile, and the motion of the basal anterior mitral leaflet was markedly restricted. The mitral valve gradient was measured at 14/29 mmHg, with a valve area of 0.9 cm², determined using the pressure half-time method. No significant mitral regurgitation was observed. Given her medical history, frail condition, and high operative risk (STS-PROM score of 6.2%), the heart team decided to treat her with TMVR. Further pre-procedural evaluation included computed tomography-guided assessment of septal bulging, the aorto-mitral angle, and mitral valve size, along with estimation of the neo-LVOT area (*Figures 1–3*). No pre-existing LVOT obstruction was detected, and the risk for LVOT obstruction after TMVR was considered low.

**Figure 4:**
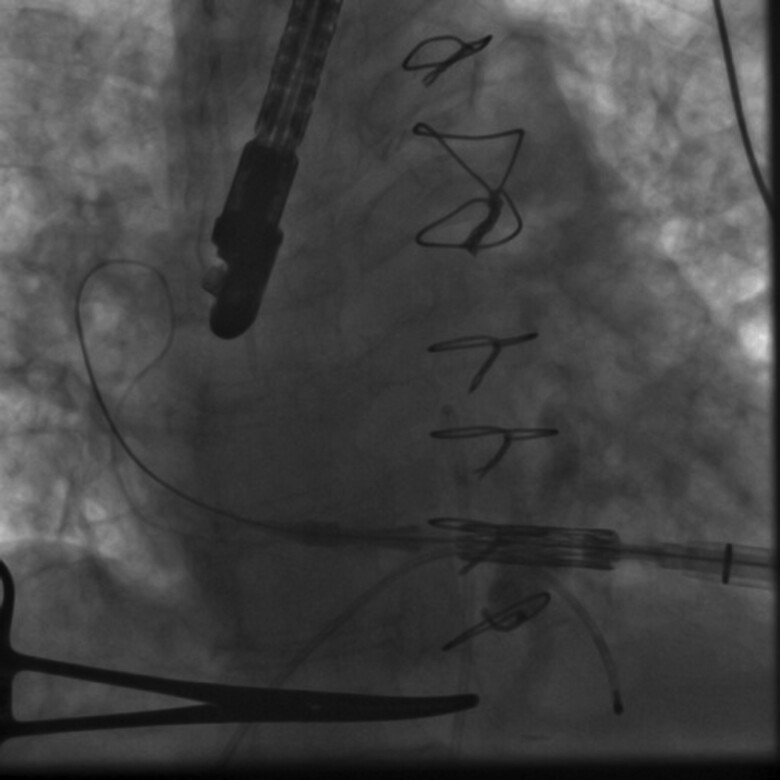
Stiff wire positioned at the posterolateral aspect of the left atrium, aligning the Edwards valve with the mitral annulus.

**Figure 5:**
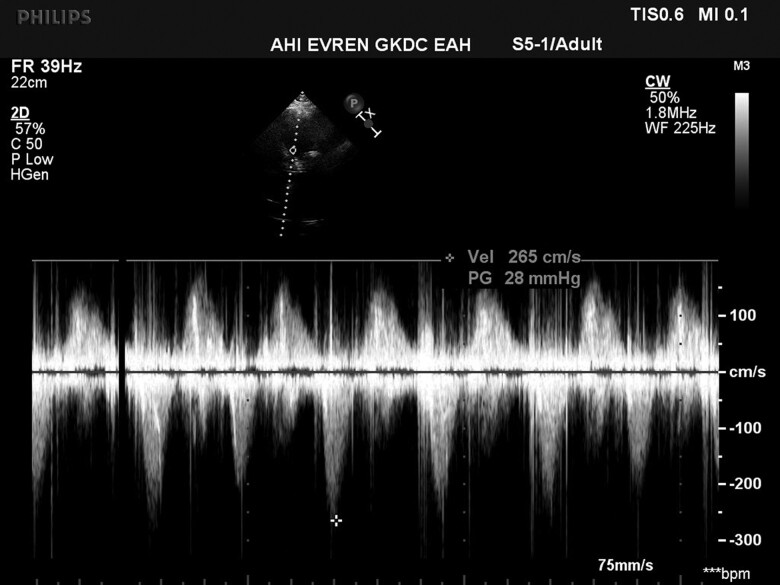
Doppler echocardiographic measured maximal gradient of 28 mmHg in the left ventricular outflow tract after the kissing balloon manoeuvre.

Transcatheter mitral valve replacement was carried out in the hybrid operating room. Following transapical puncture under general anaesthesia, a stiff wire was introduced into the left upper pulmonary vein. The SAPIEN valve was then advanced and successfully deployed in the mitral position (*Figure 4*). Post-implantation transesophageal echocardiography examination revealed an increased LVOT pressure gradient of 53/85 mmHg. Since these gradients were above acceptable levels, a kissing balloon manoeuvre was performed to alleviate the LVOT obstruction (see Supplementary material online, *Video S1*), resulting in a reduction of the LVOT peak gradient to 28 mmHg (*Figure 5*). At the post-interventional follow-up and during the subsequent hospital stay of over one week, mitral valve function was assessed as normal, and the LVOT gradient remained stable with no further increases. The patient showed no significant changes in symptoms during the short-term course, while long-term follow-up data are not available.

## Discussion

Transcatheter mitral valve replacement has evolved over the past few decades as a minimally invasive therapeutic option for treating mitral valve disease in high-risk elderly patients. However, the experience with TMVR and long-term outcomes remain limited. Transcatheter mitral valve replacement must contend with a variety of complex anatomical conditions that can result in technical challenges, such as a non-circular, saddle-shaped dynamic mitral annulus of large dimensions, irregular leaflet geometry, heterogeneous anatomy of the subvalvular apparatus, and proximity to the LVOT. The key challenges to address include effective anchoring, reliable sealing, and prevention of LVOT obstruction.^1–6^

The SAPIEN transcatheter heart valve is a low-profile, balloon-expandable valve that is commercially available for aortic valve replacement and is used off-label for TMVR. It has emerged as the predominant valve system for TMVR in the context of failing mitral bioprostheses [valve-in-valve (ViV)] and is also applied for valve-in-surgical-ring (ViR) and valve-in-mitral-annulus-calcification (ViMAC) procedures.^4–6^

Left ventricular outflow tract obstruction is a feared and potentially life-threatening complication of TMVR and has been reported in up to 10–40% of ViMAC procedures, 5% of ViR procedures, and 0.7–2% of ViV procedures.^5^ The mortality rate associated with LVOT obstruction and haemodynamic impairment in TMVR is high, reaching up to 62%.^5,6^ Major determinants of LVOT obstruction after TMVR include anterior mitral leaflet elongation, a perpendicular aorto-mitral angle, and septal hypertrophy.^2,4–9^ Several pre-emptive or bail-out strategies, such as alcohol septal ablation (ASA) and the laceration of the anterior mitral leaflet (LAMPOON) procedure, have been described to mitigate LVOT obstruction. LAMPOON involves transcatheter electromechanical splitting of the anterior mitral leaflet to move it away from the LVOT.^5,9^ While several modifications have been developed to simplify its use in certain anatomies, the technique remains challenging and has limitations; for example, it cannot be used to treat LVOT obstruction caused by the transcatheter valve skirt.^9^ Alcohol septal ablation has also been utilized as both a preventive and therapeutic strategy in selected patients, helping to reduce the gradient across the LVOT. However, significant limitations of ASA include the requirement for an adequate septal perforator artery, the risk of damaging adjacent myocardium, the potential creation of an iatrogenic ventricular septal defect, and the possibility of conduction disturbances.^10^

In our case, we employed the kissing balloon technique after identifying LVOT obstruction following the deflation of the SAPIEN transcatheter heart valve. This intervention resulted in a substantial reduction in the peak instantaneous LVOT gradient. The kissing balloon technique had previously been utilized preventively by Rahhab *et al*.^11^ to facilitate valve positioning and ensure the integrity of the neo-LVOT during TMVR. Additionally, Herrmann *et al*.^12^ described the use of simultaneous perfusion balloon inflation in the LVOT during TMVR deployment to maintain its patency. A review of the literature revealed no further applications of the kissing balloon technique in the context of TMVR.

## Conclusion

Transcatheter mitral valve replacement is a minimally invasive procedure for patients with severe mitral valve disease who are at high surgical risk. Left ventricular outflow tract obstruction is a serious complication, leading to the development of various pre-emptive and emergency strategies to address it, including the kissing balloon technique, the LAMPOON procedure, and ASA. In this case, we aim to demonstrate that the kissing balloon technique can be effectively employed as a rescue strategy for managing LVOT obstruction following TMVR.

## Supplementary Material

ytaf021_Supplementary_Data

## Data Availability

The data supporting this article are available in the article.

## Lead author biography

Dr Sibel Çatalkaya completed her medical education at the University of Ulm, Germany, followed by her residency in internal medicine and cardiology at Karl-Olga Hospital in Stuttgart. She is currently practicing at Bursa City Hospital, Turkey. Her research interests include interventional cardiology and cardiolovascular imaging, encompassing conventional and invasive echocardiography and cardiac MRI.

## Supplementary material

**Consent:** The patient provided informed consent in accordance with the COPE guidelines for this manuscript and associated images.

**Funding:** None declared.
